# Supplementary material for: AI-Driven Transcriptome Prediction in Human Pathology: From Molecular Insights to Clinical Applications
Source: Biology (Basel). 2025 Jun 4;14(6):651. doi: 10.3390/biology14060651 (PMC12189417; doi:10.3390/biology14060651)
Supplement: Supplementary file 1 [file biology-14-00651-s001.zip › biology-3576714-supplementary.pdf]

Supplementary Material

Table S1. Other AI model for predicting gene expression.

**Model Name**	**Model Type**	**Application**	**Data Type**	**Advantages**	**Limitations**
Hist2ST[148]	Transformer + GNN	Spatial transcriptomics prediction	H&E images, spatial transcriptomics data	Integrated tissue morphology and spatial gene expression	expressionLarge training data requirement, staining method sensitivity
Enformer[149]	Transformer	Gene expression prediction, enhancer-promoter interaction	Long DNA sequences (promoters/enhancers)	Long-range regulatory interaction modeling	Inter-individual prediction limitations
Xpresso[150]	Deep Learning Model	Cross-species gene expression analysis, regulation research	Genome sequences (promoter regions)	Cross-species generalization	Inferior to Enformer[149]/ Basenji2[120] in inter-individual prediction
DeepSEA[151]	Deep Convolutional Model	Regulatory variant prediction, chromatin feature analysis	Genome sequence data	Simultaneous prediction of histone modifications/TFS binding	Large training data requirement
HisToGene[152]	Deep Learning Model	Gene expression-image association research	ST data, Visium data, small-sized images	Small-sample suitability, relative generalization	Visium training difficulty, moderate clinical impact
GeneCodeR[153]	Spatial Analysis Tool	Spatial gene expression analysis	Image data, gene expression data	Rapid spatial data processing	General performance, limited scalability
DeepSpaCE[23]	CNN	Gene expression-disease association research	Image data, SGE-related data	Simple implementation, cross-dataset applicability	Moderate SGE prediction performance

EGNv1/v2[154]	GNN	Survival analysis, ST modeling	ST data, Visium data, reference datasets	Low training data requirement, clinical translation potential	Complex reference dataset planning, high computational cost
TCGN[155]	Transformer + GNN	Histopathology-based gene expression prediction, cancer subtyping	H&E images, ST data, bulk RNA-seq data	Single-spot image input, simplified workflow	Limited validation on specific datasets, architectural complexity
THItoGene[156]	Deep Learning Model	Tissue spatial feature extraction, gene expression prediction	Image data, Visium data	Multi-perspective feature analysis	Model complexity, overfitting risk, hard-coded settings
iStarST[157]	Deep Learning Model	Single-cell level gene expression prediction	Spot-based data, H&E images	High-resolution spatial gene expression inference	Code modification needed for dynamic data loading
BrST-Net[158]	EfficientNet-based	Breast cancer histopathology-gene expression prediction	H&E images, ST data, gene expression count matrices	Large-scale screening framework	Limited generalization to non-breast cancers, preprocessing complexity
